# Inflammatory markers of symptomatic remission at 6 months in patients with first-episode schizophrenia

**DOI:** 10.1038/s41537-023-00398-1

**Published:** 2023-10-04

**Authors:** Honey Kim, Seon-Hwa Baek, Ju-Wan Kim, Seunghyong Ryu, Ju-Yeon Lee, Jae-Min Kim, Young-Chul Chung, Sung-Wan Kim

**Affiliations:** 1 Department of Psychiatry, Chonnam National University Medical School, Gwangju, Korea; 2 Mindlink, Gwangju Bukgu Community Mental Health and Welfare Center, Gwangju, Korea; 3 Department of Psychiatry, Chonbuk National University Medical School, Jeonju, Korea

**Keywords:** Schizophrenia, Biomarkers

## Abstract

Neuroinflammation contributes to the pathophysiology of various mental illnesses including schizophrenia. We investigated peripheral inflammatory cytokines as a biomarker for predicting symptomatic remission in patients with first-episode schizophrenia. The study included 224 patients aged 15–60 years who fulfilled the criteria for schizophrenia spectrum disorder with a treatment duration ≤6 months. Serum levels of tumor necrosis factor (TNF) -α, interferon-γ, interleukin (IL)-1α, IL-1β, IL-6, IL-8, IL-10, and IL-12 were measured. Psychotic symptoms, depressive symptoms, and general functioning were assessed using the Positive and Negative Syndrome Scale, Beck Depression Inventory (BDI), Calgary Depression Scale for Schizophrenia, and Personal and Social Performance scale, respectively. Duration of untreated psychosis (DUP) was also recorded. We investigated the factors associated with remission for each sex in logistic regression analysis. In total, 174 patients achieved remission at the 6-month follow-up (females, 83.5%; males, 70.9%). Remission was associated with older age and lower BDI scores in male patients and with lower TNF-α levels and shorter DUP in female patients. Our findings suggest that peripheral inflammatory cytokines may impede early symptomatic remission in female patients with schizophrenia. In addition, depressive symptoms in males and long DUP in females may be poor prognostic factors for early remission in patients with first-episode psychosis.

## Introduction

Environmental risk factors may affect individuals with a genetic susceptibility to schizophrenia^1,2^. Several recent studies have investigated the effect of these risk factors on the immune system, that is, the pathophysiology of schizophrenia at the neurobiological level^2–4^. Cytokines play a significant role in the immunological process associated with the pathophysiology of schizophrenia. Cytokines are small glycoproteins that mediate communication between various immune and nerve cells. They are grouped according to function and may have pro- or anti-inflammatory effects^5^. Cytokine neuroactivity is mainly mediated by microglia, which are immune cells located in the brain^6^.

Several studies have shown an association between increased inflammatory cytokine concentrations and microglia activation in schizophrenia^7,8^. Stress-induced microglial activity releases inflammatory cytokines in individuals with a genetic predisposition, causing abnormal neurogenesis, neuronal degradation, and white matter abnormalities, all of which contribute to the pathophysiology of schizophrenia^3,4,9,10^. When exposed to harmful stimuli, such as stress, the microglia are activated and generate inflammatory cytokines including TNF-α, IL-6, IL-1β, and IFN-γ^11^.

Three major mechanisms explain the relationship between changes in inflammatory cytokines and the development of schizophrenia: proinflammatory cytokines interfere with neurodevelopment, which increases the likelihood of psychosis^12^; cytokine changes cause oxidative stress and are associated with increased neurodegeneration^13^; and changes in inflammatory cytokines promote the formation of kynurenic acid (KYNA), an endogenic antagonist of the N-methyl-D-aspartate (NMDA) receptor, which that is thought to reduce glutamatergic signaling and play a causative role in schizophrenia^14^.

Evidence suggesting that psychiatric conditions are inflammatory disorders is mounting^9,10^. Serum levels of proinflammatory cytokines, including interleukin (IL)-1, IL-6, IL-8, IL-12, interferon-γ (IFN-γ), and tumor necrosis factor-α (TNF-α), are increased in patients with depression^15,16^, and serum levels of IL-4, TNF-α, IL-1β, and IL-6 are higher in patients with bipolar disorder than in healthy controls^17,18^. Moreover, several studies have found changes in inflammatory cytokine levels in patients with schizophrenia^7,8^. A meta-analysis conducted by Miller et al.^7^, revealed that serum IL-1β, IL-6, IL-12, IFN-γ, TNF-α, transforming growth factor-β, and sIL-2R levels were significantly higher in patients with first-episode schizophrenia than in the control group. A meta-analysis of 62 studies found that the IL-1 receptor antagonist and sIL-2R, and IL-6 levels were significantly increased in patients with schizophrenia compared with controls^15^.

Thus, evidence from several studies suggests that neuroinflammation contributes to the pathophysiology of various mental illnesses. Furthermore, an association between changes in inflammatory cytokine levels and treatment response in patients with depression has been reported^19^. While several studies have examined the association between inflammatory cytokine changes and clinical manifestations (e.g., stage of chronicity, cognitive function, severity of psychotic symptoms, and suicidality) in patients with schizophrenia^20–23^, few studies have investigated the longitudinal relationship between peripheral inflammatory cytokine levels and symptomatic remission in first-episode schizophrenia.

Patients with first-episode schizophrenia who achieve remission following general psychiatric treatment have significantly different clinical and functional outcomes than those without remission^24,25^. Early remission of symptoms is associated with fewer psychotic and emotional symptoms, improved psychosocial function over the next 5 years, and a lower likelihood of recurrence after 7.5 years^25–27^. Moreover, clinical stability may be a more useful clinical indicator of schizophrenia and symptomatic remission than the subjective scales currently used^27,28^, highlighting the importance of identifying the factors associated with the remission of psychotic symptoms in patients with first-episode schizophrenia.

We hypothesized that inflammatory cytokines, which are associated with the pathophysiology of schizophrenia, negatively affect remission in patients with first-episode schizophrenia. We investigated the usefulness of peripheral inflammatory cytokine levels as a predictive biomarker of remission in patients with first-episode schizophrenia.

## Results

Our study included 224 patients (46.0% males and 54.0% females) with first-episode psychosis. The median (interquartile range) age at baseline was 25 (21–32) years. The median (interquartile range) durations of antipsychotic treatment and DUP were 1 (0.7–1.5) month and 3 (1–13.4) months, respectively. The most common diagnosis was schizophrenia (*n* = 157, 70.1%), followed by schizophreniform disorder (*n* = 42, 18.8%), other specified schizophrenia spectrum disorder (*n* = 19, 8.5%), and schizoaffective disorder (*n* = 6, 2.7%).

Supplementary Table S1 presents comparisons of cytokine levels by sex. The IFN-γ, IL-1β, and IL-12 levels were significantly higher in female patients than male patients (Supplementary Table S1). Of the total 224 patients, 174 patients (77.7%) achieved remission at the 6-month follow-up. The remission rate was significantly higher in females compared to males (83.5% vs. 70.9%, respectively; chi-square = 5.092, *p* = 0.024).

Table 1 displays the clinical characteristics and cytokine levels in relation to remission for each sex among patients with psychosis. Among male patients, the Mann–Whitney *U*-test revealed significant associations between remission at 6 months and baseline scores on PANSS (total and subscales), PSP, BDI, and CDSS. While the remission group tended to be older and had a shorter DUP, these differences did not reach statistical significance (*p* = 0.083 and *p* = 0.079, respectively). Among female patients, TNF-α, DUP, and PSP and PANSS negative, general, and total scale scores were significantly associated with remission. Diagnosis, type of antipsychotics, chlorpromazine-equivalent dosage, body mass index, smoking history, and duration of treatment were not associated with remission.

**Table 1:** Comparisons of clinical characteristics and cytokine levels according to the remission at 6 months in patients with psychosis for each sex.

		Male				Female			
	Total (*N* = 224)	Remission (+) (*N* = 73, 70.9%)	Remission (-) (*N* = 30, 29.1%)	Statistical value	*p*-value	Remission (+) (*N* = 101, 83.5%)	Remission (-) (*N* = 20, 16.5%)	Statistical value	*p*-value
*Sociodemographic and clinical characteristics*									
Age, Med, (IQR) year	25.0 (21.0–32.0)	25.0 (21.0–32.0)	23.0 (19.0–29.3)	*Z* = -1.734	0.083	26.0 (21.0–32.0)	26.5 (20.5–30.8)	*Z* = -0.297	0.767
DUP, Med. (IQR) month	3.0 (1.0–13.4)	2.0 (1.0–12.5)	7.0 (1.8–19.5)	*Z* = -1.759	0.079	3.0 (1.0–11.0)	8.5 (2.5–50.8)	*Z* = -2.730	**0.006**
Duration of Tx, Med (IQR) month	1.0 (0.7–1.5)	1.0 (1.0–1.7)	1.0 (0.6–1.6)	*Z* = -1.258	0.208	1.0 (0.6–1.5)	1.0 (0.7–1.9)	*Z* = -0.674	0.500
Inpatient status, *N* (%)	109 (48.7)	37 (72.5)	14 (27.5)	*χ*^2^ = 1.137	0.711	49 (84.5)	9 (15.5)	*χ*^2^ = 0.083	0.774
Smoking history, *N* (%)	77 (35.8)	43 (76.8)	13 (23.2)	*χ*^2^ = 2.078	0.149	21 (84.0)	4 (16.0)	*χ*^2^ = 0/991	0.991
CPZ eq. dosage	400 (200–619)	400 (200–675)	450 (238–850)	*Z* = -0.963	0.336	300 (138–600)	400 (156–619)	*Z* = -0.494	0.621
BMI, Med. (IQR) m^2^	21.6 (19.7–24.0)	23.3 (20.8–26.1)	23.2 (20.1–24.5)	*Z* = -0.581	0.561	20.9 (19.4–22.8)	21.2 (19.0–23.0)	*Z* = -0.227	0.821
Diagnosis, *N* (%)				*χ*^2^ = 2.304	0.311			*χ*^2^ = 1.404	0.496
Schizophrenia	157 (70.1)	51 (69.9)	22 (30.1)			68 (81.0)	16 (19.0)		
Schizophreniform	42 (18.8)	15 (83.3)	3 (16.7)			21 (87.5)	3 (12.5)		
Others	25 (11.2)	7 (58.3)	5 (41.7)			12 (92.3)	1 (7.7)		
Antipsychotics, *N* (%)				*χ*^2^ = 1.983	0.576			*χ*^2^ = 2.497	0.476
Amisulpride	55 (25.0)	23 (69.7)	10 (30.3)			18 (90.0)	2 (10.0)		
Paliperidone	102 (46.4)	35 (74.5)	12 (25.5)			46 (83.6)	9 (16.4)		
Aripiprazole	42 (19.1)	4 (50.0)	4 (50.0)			30 (88.2)	4 (11.8)		
Others	23 (10.5)	9 (69.2)	4 (30.8)			7 (70.0)	3 (30.0)		
*Cytokines*									
Tumor Necrosis Factor-α	8.8 (7.0–12.3)	9.2 (7.3–13.3)	8.8 (7.3–10.9)	*Z* = -0.788	0.431	8.5 (6.6–10.8)	9.9 (8.6–18.9)	*Z* = -2.551	**0.011**
Interleukin-1b	2.5 (1.7–3.6)	2.1 (1.6–3.5)	2.1 (1.1–3.5)	*Z* = -0.610	0.542	2.6 (1.9–3.7)	3.1 (2.1–6.1)	*Z* = -1.521	0.128
Interleukin-6	3.8 (2.4–5.7)	3.5 (2.5–5.3)	3.0 (1.7–6.8)	*Z* = -0.730	0.466	4.0 (2.5–5.8)	3.8 (2.2–5.8)	*Z* = -0.830	0.406
Interleukin-8	5.0 (3.2–8.8)	4.6 (3.1–10.3)	5.3 (3.1–9.6)	*Z* = -0.240	0.811	5.1 (3.4–9.3)	4.0 (2.9–6.6)	*Z* = -1.549	0.121
Interleukin-10	13.9 (6.4–22.1)	13.9 (5.6–22.8)	11.7 (6.0–18.5)	*Z* = -1.067	0.286	15.2 (8.6–23.1)	9.0 (2.2–23.7)	*Z* = -1.521	0.128
Interleukin-12	5.1 (3.8–7.9)	4.6 (3.5–7.3)	4.4 (2.8–6.8)	*Z* = -1.009	0.313	5.5 (4.3–8.1)	6.5 (3.6–17.7)	*Z* = -0.377	0.706
Interferon-γ	21.7 (12.0–29.6)	20.9 (10.5–28.1)	16.7 (11.0–23.1)	*Z* = -1.029	0.303	25.0 (16.0–31.5)	17.2 (9.1–35.4)	*Z* = -1.290	0.197
*Psychiatric measures*									
PANSS, Positive, Med (IQR)	15.0 (12.0–18.0)	14.0 (12.0–17.5)	16.5 (14.0–21.0)	*Z* = -3.080	**0.002**	15.0 (12.0–18.0)	17.0 (13.5–19.5)	*Z* = -1.459	0.145
Negative, Med (IQR)	17.0 (14.0–20.0)	15.0 (13.0–19.0)	19.0 (16.8–21.3)	*Z* = -2.738	**0.006**	16.0 (14.0–19.5)	20.0 (18.0–23.0)	*Z* = -3.454	**0.001**
General, Med (IQR)	34.0 (30.0–39.0)	32.0 (28.0–37.0)	37.5 (33.8–41.0)	*Z* = -2.937	**0.003**	34.0 (29.0–38.5)	39.0 (33.5–46.3)	*Z* = -2.872	**0.004**
Total, Med (IQR)	67.5 (57.0–76.0)	63.0 (55.0–74.5)	70.5 (67.5–81.0)	*Z* = -3.442	**0.001**	66.0 (56.0–73.0)	74.5 (71.3–82.3)	*Z* = -3.438	**0.001**
PSP, Med (IQR)	62.0 (53.0–65.0)	63.0 (54.3–65.0)	55.0 (49.5–62.0)	*Z* = -2.348	**0.019**	63.0 (55.0–70.0)	55.0 (48.5–61.8)	*Z* = -3.080	**0.002**
BDI, Med (IQR)	7.0 (2.0–15.0)	6.0 (1.3–12.8)	13.0 (5.0–25.3)	*Z* = -3.296	**0.001**	7.0 (2.0–15.0)	6.0 (2.3–28.8)	*Z* = -0.229	0.819
CDSS, Med (IQR)	3.0 (1.0–7.0)	2.0 (0.0–6.0)	5.0 (1.8–9.0)	*Z* = -2.284	**0.022**	3.0 (1.0–8.0)	3.0 (1.0–8.0)	*Z* = -0.586	0.558

Abbreviations: *Med* Median, *IQR* Interquartile Range, *SD* Standard Deviation, *Tx* Treatment, *CPZ* eq. Chlorpromazine-equivalent, *DUP* Duration of Untreated Psychosis, *PANSS* Positive and Negative Syndrome Scale, *PSP* Personal and Social Performance, *BDI* Beck Depression Inventory, *CDSS* Calgary Depression Scale for Schizophrenia. All *p*-values are empirical. Bold values denote statistical significance at the *p* < 0.05 level.

Multivariate logistic regression analysis results, shown in Table 2, indicate that a higher potential for symptomatic remission in men was associated with older age (*p* = 0.042) and lower baseline BDI scores (*p* = 0.013). In contrast, for women, shorter DUP (*p* = 0.025) and lower baseline peripheral TNF-*α* levels (*p* = 0.006) were associated with a higher likelihood of symptomatic remission (Table 2).

**Table 2:** Logistic regression analysis to predict factors associated with remission at 6 months in patients with psychosis for each sex.

Factors	Male			Female		
	B	OR^*^ (95% CI)	*p*-value	B	OR^* (^95% CI)	*p*-value
Age, year	4.624	101.90 (1.18–8776.15)	**0.042**			
DUP, month	-0.469	0.626 (0.242–1.620)	0.334	-1.326	0.265 (0.083–0.844)	**0.025**
PANSS, positive	-5.198	0.006 (0.000–2.070)	0.086			
PANSS, negative	0.674	1.963 (0.096–40.248)	0.662	-4.930	0.007 (0.000–12.365)	0.194
PANSS, general psychopathology	-2.250	0.105 (0.000–499.652)	0.655	-3.754	0.023 (0.000–59.712)	0.348
Personal and Social Performance scale	-0.009	0.991 (0.018–54.227)	0.961		0.054 (0.000–142.452)	0.467
Beck Depression Inventory	-2.068	0.126 (0.025–0.647)	**0.013**			
Calgary Depression Scale for Schizophrenia	0.464	1.591 (0.299–8.479)	0.586			
Tumor Necrosis Factor-α		–	–	-4.224	0.015 (0.001–0.307)	**0.006**

Abbreviations: *OR* Odds Ratio, *DUP* Duration of Untreated Psychosis, *PANSS* Positive and Negative Syndrome Scale.

Bold values denote statistical significance at the *p* < 0.05 level.

## Discussion

Little is known about the relationship between symptomatic remission and inflammatory markers in patients with schizophrenia. We investigated the relationship between peripheral inflammatory cytokine levels and symptomatic remission in patients with first-episode schizophrenia. We found that baseline peripheral TNF-α levels and DUP were significantly negatively associated with remission at the 6-month follow-up in females, whereas lower baseline BDI scores and older age were significantly associated with a higher probability of remission in males. Our findings suggest that peripheral inflammatory cytokines may impede early symptomatic remission in patients with psychosis.

In our study, 77.7% of the participants achieved symptomatic remission at the 6-month follow-up. This finding is consistent with previous studies showing a good treatment response in first-episode schizophrenia^29,30^. The female patients in our study had a higher remission rate than the males, consistent with previous reports^31,32^. In a 2-year follow-up study of 200 community-based participants with schizophrenia, remission rates were higher in females than in males^31^. In a cohort study of 533 patients with first-episode psychosis, remission and recovery rates were higher in female participants at the 1-year follow-up^32^. Several factors contribute to the association between female gender and higher remission rates including better responses to medication, a protective effect of estrogen (which can reduce dopamine concentrations), positive attitudes toward medication use and help-seeking behavior, and better access to social networks that support treatment compliance^32–34^. More research is needed to fully understand the factors underlying sex-based differences in schizophrenia remission rates to help identify at-risk groups and provide effective interventions.

Several studies have shown that higher levels of inflammation, characterized by elevated proinflammatory cytokine concentrations, are associated with poor clinical outcomes in patients with schizophrenia^35,36^. We found that remission at the 6-month follow-up was negatively associated with baseline peripheral TNF-α levels in female patients. TNF-α is an important proinflammatory cytokine produced in neurons and glial cells. Several studies have shown that peripheral TNF-α levels are increased in patients with first-episode schizophrenia^37–39^, and TNF-α-mediated immune-neurotoxicity contributes to cognitive impairment and the overall severity of schizophrenia^36,40^. TNF-α levels are positively associated with adverse symptoms and negatively correlated with various cognitive functions, including attention span, verbal memory, and executive function in patients with first-episode schizophrenia^21,41^. The TNF-α–238 G/A polymorphism is associated with treatment resistance and risk of suicide attempts in patients with schizophrenia^42^. A crossover study investigating cytokine levels in 54 patients with schizophrenia found that TNF-*α* expression tended to be increased in patients with treatment-resistance schizophrenia, but the difference was not statistically significant^43^.

We found that proinflammatory cytokines had a negative effect on treatment response and remission in female, but not male, patients with schizophrenia, whereas a previous study found no significant sex differences in peripheral cytokine levels^44^. O’Connell et al.^45^ reported that TNF-α levels were significantly increased in female, but not male, patients with schizophrenia. The response to immune stimuli tends to be stronger in females than in males, which has short-term benefits for the acute immune response^46,47^. However, continuous activation of the immune system, as occurs in chronic diseases, is harmful, and males may have increased sensitivity and vulnerability to chronic immune activation^48^. Differences in sex-related immune responses are thought to result from differences in sex chromosome genes and hormones, including estrogen and progesterone^49^. Thus, our findings may be explained by differences in immunological characteristics between females and males.

We found that shorter DUP was associated with a higher likelihood of remission in female patients. Several studies have shown that shorter DUP is significantly correlated with better outcomes in terms of symptom remission, functional recovery, and quality of life in individuals with first-episode psychosis^29,50^. Longer DUP is associated with lower remission rates, poor premorbid functioning, insidious onset of disease, higher severity and persistence of psychotic symptoms (negative symptoms, in particular), and delayed treatment^51,52^. DUP is influenced by patient and guardian factors related to help-seeking (e.g., stigmatization or misunderstanding of psychotic illnesses) and factors related to the care providers (e.g., physicians, counselors, teachers, religious personnel, mental health service specialists) in the institutions where the first contact is made (e.g., ability of case managers to identify psychosis or access to early intervention services)^53–56^. Therefore, it is crucial that government authorities and health providers deliver evidence-based interventions that reduce the DUP and improve clinical outcomes in first-episode psychosis.

In our study, lower baseline BDI scores were associated with a higher likelihood of remission in male patients. Previous studies in patients with schizophrenia found that baseline depression severity was negatively correlated with remission after treatment for 1 year^57^ and that alleviation of depressive symptoms increased the likelihood of remission^27^, consistent with our findings. Patients with first-episode psychosis and co-occurring depression have poorer treatment outcomes (e.g., more severe symptoms, longer hospitalization, poorer functioning and quality of life, lower remission rates, and a higher risk of psychotic relapse and suicide) than patients without depression^58–60^. Depressive symptoms correlate with positive and negative symptoms in patients with first-episode psychosis^61^. Our criteria for severity and symptomatic remission were based on the positive and negative symptoms of schizophrenia, which may have affected our findings. Moreover, patients with first-episode psychosis and depression have a poorer response to antipsychotic medication and poorer medication adherence than those without depression^62,63^. Therefore, remission rates in patients with first-episode psychosis can be improved by assessing and treating depressive symptoms. A better understanding of the relationship between depressive symptoms and symptomatic remission is essential for developing a comprehensive intervention strategy.

We found that older age was associated with a higher likelihood of remission in male patients. Schizophrenia typically develops in early adulthood, i.e., at 15–25 years of age. Onset at an earlier age is associated with a poor therapeutic response to antipsychotics, poor prognosis, more severe symptoms, poor cognitive performance, and social withdrawal, and is generally more difficult to treat^64–66^. A review of multicenter studies involving >100 mental health facilities found a positive correlation between age at onset and the likelihood of remission in patients with schizophrenia^67^ highlighting the importance of providing effective psychiatric treatment for this vulnerable population.

Our findings support the potential use of anti-inflammatory drugs as an adjunctive treatment for patients with schizophrenia. Notable adjunctive therapies for schizophrenia include omega-3 fatty acids, minocycline, aspirin, and N-acetylcysteine (NAC). Several meta-analyses examining these drug types have reported reductions in the overall severity of symptoms in patients with schizophrenia, suggesting their potential utility as augmentation therapies alongside antipsychotics^68–72^. Recent meta-analyses have provided compelling quantitative evidence, demonstrating significant improvements in cognitive domains like executive function, visual learning, and attention following augmentation with minocycline or pregnenolone^73,74^ While the investigation of anti-inflammatory drugs as a novel treatment for schizophrenia is ongoing, it’s important to note that the existing data are limited in terms of both quality and quantity. Therefore, further research is necessary.

Although the use of anti-inflammatory drugs as a novel treatment for schizophrenia is under investigation, the data are limited by the quality and quantity of the studies; thus, further research is needed. Building on previous findings, we propose using anti-inflammatory drugs that target inflammatory cytokines to achieve symptomatic remission in patients with schizophrenia. This approach shows promise for symptomatic remission in patients who exhibit changes in cytokine levels; however, additional research is needed.

Our study has several limitations. First, some of our participants were drug-naïve, and it is possible that the antipsychotics prescribed to these patients affected their inflammatory cytokine levels^75^. A previous study in drug-naïve patients with first-episode schizophrenia also found changes in inflammatory cytokine levels^7^; thus, further research is needed to clarify the relationship between alterations in inflammatory cytokine levels and antipsychotic use in patients with schizophrenia. Second, even though there were no significant differences between the types of antipsychotics and remission rates, it’s possible that different antipsychotics exert varying influences on symptomatic remission. Therefore, further studies are warranted to compare the trajectory of remission among different antipsychotics. Third, we measured peripheral cytokine levels, which do not directly reflect the immunoinflammatory state of the central nervous system. Because they are easy to measure, peripheral cytokine levels are generally used in studies; however, more research is needed to determine the extent to which peripheral cytokine levels reflect changes in brain concentrations. Finally, the potential type I errors should be considered because this study used empirical *p*-values instead of correcting for multiple testing. Nevertheless, our results provide a basis for future investigations into the roles of specific proinflammatory cytokines and novel treatment strategies.

In conclusion, our findings suggest that peripheral TNF-α levels may serve as a predictor of poor response to antipsychotic treatments in females with first-episode schizophrenia spectrum disorder. This could contribute to enhancing our understanding of the role of inflammation in the course of schizophrenia. Additionally, we observed that depressive symptoms in males and a longer DUP in females may impede early remission in patients with first-episode psychosis. Further research is needed to investigate whether modifying these factors can improve the clinical course of psychosis.

## Methods

### Study design

We designed a longitudinal cohort study to predict remission at 6-month follow-up. Data were obtained from an early psychosis cohort enrolled in the Gwangju Early Treatment and Intervention Team (GETIT) cohort study, which included patients with a recent-onset psychotic disorder. Patients with an antipsychotic treatment duration >6 months were excluded to avoid the confounding effects of long-term antipsychotic medication. All patients aged 15–60 years met the criteria for schizophrenia spectrum disorder and other psychotic disorders, according to the Diagnostic and Statistical Manual of Mental Disorders, Fifth Edition^76^. Patients with a substance- or medication-induced psychotic disorder, psychotic disorder due to another medical condition, or severe neurological or medical illness were excluded. Our study was conducted between January 2015 and October 2022 and was approved by the Chonnam National University Hospital Institutional Review Board (CNUH-2014-225). All patients provided written informed consent before participation.

### Demographic and clinical measures

Baseline sociodemographic and clinical data included age, sex, education level, body mass index, smoking history, type of antipsychotics, diagnosis, duration of untreated psychosis (DUP), and duration of treatment. The DUP is defined as the period from the emergence of psychotic symptoms to the initiation of effective antipsychotic treatment, typically characterized by the continuous use of antipsychotic medication use for a minimum of 2 months or a duration that results in clinically significant improvement^77^. The prescribed antipsychotic doses were converted to the chlorpromazine equivalent^78^.

Psychotic symptoms were assessed using the Korean version of the Positive and Negative Syndrome Scale (PANSS). The Cronbach’s alpha coefficients for the positive, negative, and general psychopathology subscales were 0.73, 0.84, and 0.74, respectively, indicating acceptable internal consistency across all three subscales of the Korean version of the PANSS^79,80^. We used the remission criteria proposed by Andreasen^81^. Patients were considered to have achieved symptomatic remission if they obtained a score ≤3 on each of the following PANSS items: P1, P2, P3, N1, N4, N6, G5 and G9. General functioning was measured using the Korean version of the Personal and Social Performance (K-PSP) scale^82,83^. The internal consistency of the K-PSP was acceptable (Cronbach’s *α* = 0.79)^83^. We assessed depressive symptoms using the Korean version of the Beck Depression Inventory (BDI)^84,85^ and Calgary Depression Scale for Schizophrenia (CDSS)^86–88^. The Cronbach’s alpha for the K-BDI, and K-CDSS were 0.880, and 0.852, respectively, indicating favorable internal consistency^85,86^. The K-BDI and K-CDSS showed good validity, demonstrating strong correlations with other depression scale^85,86^.

### Cytokine measurements

A venous blood sample was obtained from patients on the same day as the baseline clinical assessments. All samples were processed within 2 h of blood collection and stored at -80 °C until further analysis. We quantitatively determined the steady-state levels of the circulating inflammatory cytokines of interest, including TNF-α, IFN-γ, IL-1β, IL-6, IL-8, IL-10, and IL-12. Plasma concentrations were determined using a human cytokine/chemokine magnetic bead panel (Milliplex MAP Kit; Millipore Corp., Billerica, MA, USA) according to the manufacturer’s instructions. A Luminex Bio-Plex 100 Analyzer (MAGPIX; Luminex Corp., Austin, TX, USA) was used to identify individual microspheres, and the results were quantified based on fluorescent reporter signals using Milliplex Analyst 5.1 (Merck KGaA, Darmstadt, Germany) and Luminex xPONENT (Luminex Corp.) acquisition software. We assessed the median fluorescence intensity using a five-parameter logistic or spline curve-fitting method to calculate the cytokine concentrations in each sample. A laboratory company (GC Cell, Yongin, Korea) performed all assays and quality control procedures according to the manufacturer’s instructions.

### Statistical analysis

The participants were divided into two groups according to remission at 6-month follow-up. Differences in cytokine levels according to sex were compared using the Mann–Whitney *U-*test. Demographic and clinical characteristics, including cytokine levels, were compared according to remission status for each sex using the Mann–Whitney *U*-test or chi-squared test. Parameters that were associated with remission at 6 months in univariate analyses (*p* < 0.1) were entered into a logistic regression analysis after log transformation to adjust for confounding effects. All statistical tests were two-tailed, and *p*-values < 0.05 were considered to indicate statistical significance. The statistical analysis was performed using SPSS software (version 25.0; IBM Corp., Armonk, NY, USA).

### Supplementary information

Table S1.

## Data Availability

The data will be made available upon request.
