# Supplementary material for: Inflammatory markers of symptomatic remission at 6 months in patients with first-episode schizophrenia
Source: Schizophrenia (Heidelb). 2023 Oct 4;9(1):68. doi: 10.1038/s41537-023-00398-1 (PMC10550944; doi:10.1038/s41537-023-00398-1)
Supplement: Supplementary file 1 — Table S1. [file 41537_2023_398_MOESM1_ESM.docx]

Table S1. Comparisons of cytokine levels by sex

|  | Female | Male | Z | p-value |
| --- | --- | --- | --- | --- |
| Tumor Necrosis Factor-α | 8.6 (6.7-12.1) | 9.1 (7.3-12.4) | -1.044 | 0.297 |
| Interleukin-1b | 2.8 (1.9-3.7) | 2.1 (1.5-3.5) | -2.552 | 0.011 |
| Interleukin-6 | 3.9 (2.5-5.8) | 3.4 (2.2-5.6) | -0.908 | 0.364 |
| Interleukin-8 | 5.0 (3.4-8.8) | 4.8 (3.1-9.2) | -0.066 | 0.947 |
| Interleukin-10 | 14.8 (7.0-23.1) | 13.4 (5.9-20.5) | -1.363 | 0.173 |
| Interleukin-12 | 5.5 (4.2-8.4) | 4.5 (3.3-7.3) | -2.317 | 0.033 |
| Interferon-γ | 24.4 (14.5-32.2) | 19.4 (10.7-25.5) | -2.559 | 0.011 |

Values are median (interquartile range).
